# Variations in Psychopathy Dimensions Among Incarcerated Individuals Convicted of Violent Offenses in Romania: The Role of Offense Type, Age, and Personality Traits

**DOI:** 10.3390/bs16101933

**Published:** 2026-10-17

**Authors:** Andreea-Cătălina Forțu, Cornelia Rada

**Affiliations:** 1 Personnel Psychology Service, National Administration of Penitentiaries, 051543 Bucharest, Romania; andreea_catallina@yahoo.com; 2 Department of Psychology, “Constantin Rădulescu-Motru” Institute of Philosophy and Psychology, School of Advanced Studies of The Romanian Academy, Romanian Academy, 050711 Bucharest, Romania; 3 Biomedical Department, Francisc I. Rainer Institute of Anthropology, Romanian Academy, 050711 Bucharest, Romania

**Keywords:** psychopathy, personality traits, violent behavior, incarcerated individuals, agreeableness and conscientiousness

## Abstract

Psychopathy is a multidimensional construct consistently associated with antisocial and violent behavior; however, its affective–interpersonal and behavioral components may contribute differently to distinct forms of violent offending. The present study investigated variations in psychopathy dimensions according to the type of violent offense and age, as well as the predictive role of agreeableness and conscientiousness in a sample of incarcerated individuals convicted of violent crimes. The sample comprised 500 incarcerated individuals from six Romanian prisons (M age = 38.49 years, SD = 10.39), assessed using the Self-Report Psychopathy Scale–III (SRP-III), the Levenson Self-Report Psychopathy Scale (LSRP), and the Agreeableness and Conscientiousness dimensions of the Big Five personality model. Data were analyzed using analyses of variance (ANOVA), Pearson correlation analyses, and hierarchical linear regression. The results revealed significant differences in psychopathy levels according to the type of violent offense (SRP-III: *F*(2, 484) = 12.13, *p* < 0.001, η^2^ = 0.048), with higher psychopathy scores observed among individuals convicted of physical violence offenses. Psychopathy decreased significantly with age, particularly in its behavioral dimension. Agreeableness and conscientiousness emerged as significant negative predictors of psychopathy (β = −0.179, *p* < 0.001, and β = −0.175, *p* = 0.001, respectively), accounting for an additional 10% of the explained variance. These findings support a dimensional conceptualization of psychopathy and highlight the role of personality traits in the psychological profile of violent offenders. The results have important implications for violence risk assessment and the development of individualized intervention strategies within correctional settings, although the modest proportion of variance explained (13%) and the predominantly small effect sizes observed indicate that these findings should be considered preliminary rather than a basis for standalone clinical decision-making.

## 1. Introduction

Psychopathy is one of the most extensively studied constructs in criminological psychology and has been consistently associated with antisocial behavior, violence, and persistent criminal trajectories (Hare, 2003; Leistico et al., 2008). Characterized by deficits in empathy, interpersonal manipulation, and heightened impulsivity, psychopathy is currently conceptualized as a multidimensional construct encompassing affective, interpersonal, and behavioral components (Hare & Neumann, 2008).

Contemporary conceptualizations of psychopathy increasingly emphasize its heterogeneous and multidimensional nature. Rather than representing a unitary personality configuration, psychopathy is currently understood as a constellation of interrelated affective, interpersonal, behavioral, and antisocial characteristics that may vary considerably across individuals and contexts (De Brito et al., 2021; Sellbom & Drislane, 2021). This perspective is reflected in contemporary multifactorial models, which suggest that distinct psychopathy dimensions are differentially associated with aggression, criminal behavior, emotional processing, and social functioning.

From this perspective, assessing psychopathy solely through global scores may obscure important differences among its constituent dimensions. Recent research suggests that affective–interpersonal traits, such as lack of empathy, emotional detachment, and manipulative tendencies, contribute differently to criminal behavior than lifestyle and antisocial dimensions, which are characterized by impulsivity, poor behavioral regulation, and sensation seeking (Hamilton & Newman, 2018; Thomson et al., 2025).

Accordingly, a dimensional approach to psychopathy provides a more nuanced understanding of violent behavior and facilitates the identification of the psychological mechanisms underlying different forms of violent offending. This perspective is further supported by recent studies conducted among incarcerated populations, demonstrating that distinct psychopathy facets are differentially associated with difficulties in emotional processing (Ramos et al., 2026).

Given the multidimensional nature of psychopathy, recent studies have recommended the use of complementary assessment approaches capable of capturing both the affective–interpersonal and behavioral manifestations of the construct. Different self-report instruments emphasize partially distinct dimensions of psychopathy, thereby providing a more comprehensive understanding of psychopathic traits in correctional populations (Tsang et al., 2018; Međedović, 2024).

However, the existing literature has not reached a clear consensus regarding the relative contribution of psychopathy dimensions to the explanation of violent behavior (J. L. Skeem et al., 2011). Some studies have emphasized the role of affective–interpersonal characteristics, such as lack of empathy and callousness (Blair, 2005), whereas others have highlighted the contribution of behavioral dimensions, including impulsivity and an antisocial lifestyle, to the association between psychopathy and violence (Leistico et al., 2008). These inconsistent findings underscore the importance of examining psychopathy dimensions separately in relation to different forms of violent behavior.

Another limitation of the existing literature is the tendency to focus on specific psychopathy components without consistently integrating them within particular criminological contexts (Cooke & Michie, 2001). This limitation is especially relevant in correctional settings, where institutional and contextual factors may influence the expression of psychopathic traits and related behaviors (Walters, 2003). Consequently, investigating psychopathy from an integrative perspective that considers variations according to offense type and institutional context is essential.

Age has consistently been identified as a factor associated with both psychopathic traits and antisocial behavior. Research indicates that the behavioral manifestations of psychopathy, such as impulsivity, risk-taking, and an antisocial lifestyle, tend to decline across adulthood, whereas affective and interpersonal dimensions remain relatively stable (Blonigen, 2010). This distinction is consistent with contemporary models of psychopathy, which conceptualize the construct as comprising distinct affective–interpersonal and behavioral components (Hare & Neumann, 2008).

These findings support the view that psychopathy does not manifest uniformly across the lifespan and highlight the importance of investigating age-related differences in correctional populations. Examining psychopathy dimensions across age groups may contribute to a better understanding of the psychological mechanisms underlying violent behavior and adaptation to the correctional environment.

At the same time, the role of personality traits in explaining variation in psychopathy within correctional populations remains relatively underexplored. Research integrating the Big Five model has shown that low levels of agreeableness and conscientiousness are associated with antagonistic interpersonal styles and deficits in behavioral regulation (Lynam & Miller, 2019; Ross et al., 2004), and these associations have also been documented in correctional populations (Rada et al., 2025). However, the contribution of these personality traits to the explanation of psychopathy, beyond criminological variables such as offense type and sentence severity, remains insufficiently investigated in correctional populations.

Among the Big Five personality dimensions, agreeableness and conscientiousness have demonstrated the most consistent and robust associations with psychopathic traits in both community and forensic samples. Evidence synthesized in meta-analyses and systematic reviews indicates that psychopathy is characterized by a combination of antagonism, reflected in low agreeableness, and deficits in behavioral regulation, reflected in low conscientiousness (Decuyper et al., 2009; Lynam & Miller, 2019).

Low levels of agreeableness are associated with hostility, reduced empathy, interpersonal exploitation, and antagonistic social relationships, characteristics that largely overlap with the affective–interpersonal dimensions of psychopathy. In contrast, low conscientiousness is associated with impulsivity, poor self-control, irresponsibility, and behavioral disinhibition, traits that characterize the lifestyle and antisocial dimensions of psychopathy (Ross et al., 2004; Van Til et al., 2022). Consequently, focusing on these two personality dimensions provides a solid theoretical framework for understanding individual differences in psychopathic traits among violent offenders.

In this context, examining the role of agreeableness and conscientiousness is particularly important in correctional populations, where identifying the psychological factors associated with psychopathic traits may contribute to the development of tailored assessment and intervention strategies.

This issue is especially relevant in national contexts where empirical research on psychopathy in correctional settings remains limited. In Romania, violent offenses account for a substantial proportion of criminal convictions, underscoring the importance of understanding the psychological mechanisms underlying this type of offending. According to data published by the Romanian National Administration of Penitentiaries (2025), more than 60% of incarcerated individuals have been convicted of violent offenses, highlighting the social and institutional relevance of this population.

The selection of individuals convicted of violent offenses as the target population is justified not only by the serious social impact of these crimes but also by their frequent association with psychopathic traits and difficulties in institutional adjustment. Within this context, investigating psychopathy dimensions among violent offenders provides an appropriate framework for understanding the psychological mechanisms underlying violent behavior and for informing differentiated, evidence-based intervention strategies.

Although psychopathy has been extensively investigated in international forensic research, its assessment within Romanian correctional settings has received considerably less attention. Consequently, empirical evidence regarding the distribution and manifestation of psychopathic traits among incarcerated individuals in Romania remains limited. Expanding research in this area is particularly important given the high proportion of individuals convicted of violent offenses within the Romanian correctional system and the increasing emphasis on evidence-based psychological assessment and intervention.

At the national level, there is a substantial lack of studies systematically investigating psychopathy dimensions in samples drawn from multiple correctional facilities using rigorous quantitative designs and standardized psychometric instruments. Such an approach enables not only the differentiated examination of affective–interpersonal and behavioral factors but also the assessment of their relationships with violent behavior through robust statistical models. In this way, the present study contributes to strengthening the empirical evidence required for the development of differentiated psychological interventions tailored to the personality profiles of violent offenders and for identifying specific predictors of aggression within correctional settings.

Despite the considerable body of research examining the relationship between psychopathy and violent behavior, several important gaps remain. First, many studies continue to rely on global psychopathy scores, potentially overlooking meaningful differences between affective–interpersonal and behavioral dimensions. Second, the contribution of personality traits, particularly agreeableness and conscientiousness, beyond the influence of criminological variables, remains insufficiently explored in correctional populations. Third, empirical evidence from the Romanian correctional system remains limited, particularly studies based on large, multi-site prison samples. Addressing these gaps may contribute to a more nuanced understanding of psychopathy among violent offenders and support the development of empirically informed assessment and psychological intervention practices.

Although the remaining Big Five dimensions have also been examined in relation to antisocial behavior, meta-analytic and theoretical work indicates that Agreeableness and Conscientiousness show the most consistent and robust associations with psychopathy, corresponding respectively to the antagonism and disinhibition components considered central to contemporary trait-based conceptualizations of the construct (Lynam & Miller, 2019; Decuyper et al., 2009). This relative specificity justifies prioritizing these two dimensions in the present study, without implying that the remaining Big Five traits are irrelevant to psychopathy and violent behavior.

Based on these considerations, the present study aimed to investigate variations in the affective–interpersonal and behavioral dimensions of psychopathy according to the type of violent offense and age, as well as the contribution of the personality traits of agreeableness and conscientiousness to the prediction of psychopathy levels in a sample of incarcerated individuals convicted of violent offenses in Romania. Through this approach, the study seeks to refine explanatory models of psychopathy within criminological contexts and to provide practical implications for psychological assessment and intervention in correctional settings.

### Hypotheses

**Hypothesis** **1.** *The affective–interpersonal and behavioral dimensions of psychopathy were expected to vary significantly according to the type of violent offense committed, with higher levels of the behavioral dimensions expected among individuals convicted of offenses involving direct physical violence.*

**Hypothesis** **2.** *The behavioral dimensions of psychopathy were expected to be lower among incarcerated individuals in older age groups than among those in younger age groups.*

**Hypothesis** **3.** *Agreeableness and conscientiousness were expected to contribute significantly to explaining psychopathy levels after controlling for sociodemographic and criminological variables.*

## 2. Materials and Methods

### 2.1. Participants

The study was conducted on a sample of 500 incarcerated individuals convicted of violent offenses, recruited from six correctional facilities in Romania. Participants ranged in age from 21 to 74 years (M = 38.49, SD = 10.39). The sample comprised 475 men (95.0%) and 25 women (5.0%). Participants were selected based on the following inclusion criteria: conviction for a violent offense, age of at least 21 years, and provision of informed consent to participate in the study.

The sample was obtained using a purposive sampling strategy, including correctional facilities with diverse institutional profiles, such as large-capacity prisons, a prison hospital, and a correctional facility exclusively for female inmates, in order to capture the diversity of correctional settings in Romania.

The sample size was determined by the number of incarcerated individuals who met the inclusion criteria and agreed to participate during the data collection period, resulting in a final sample of 500 participants recruited from six correctional facilities.

The age groups used in the statistical analyses were established based on Blonigen’s (2010) recommendations regarding age-related variations in antisocial behavior and psychopathic traits across adulthood. Accordingly, participants were classified into four age categories: 21–30 years, 31–40 years, 41–50 years, and over 50 years. This categorization allowed the differentiation of young, middle-aged, and older adulthood, developmental stages associated with progressive changes in impulsivity, behavioral control, and engagement in antisocial behavior (Hare, 2003).

Sentence length was included as a criminological indicator reflecting both the severity of the judicial response and the seriousness of the offenses for which participants had been convicted. For statistical analyses, sentence length was categorized into three groups: short sentences (8–60 months), medium sentences (61–120 months), and long sentences (more than 120 months), facilitating comparisons among individuals serving different periods of incarceration.

### 2.2. Procedure

Data collection was conducted between May 2024 and June 2025 in accordance with the institutional regulations governing research involving incarcerated individuals. Participants were informed about the purpose of the study, the voluntary nature of participation, and the confidentiality of their responses.

The questionnaires were administered by psychologists employed within the participating correctional facilities. Data collection was carried out using paper-and-pencil questionnaires administered either in housing units or in designated areas for social reintegration activities within the correctional facilities. Participants completed the questionnaires individually. Standardized instructions were provided before questionnaire administration, and the researcher was present throughout the procedure to clarify any procedural questions without influencing participants’ responses.

Potential participants were identified through the correctional system’s electronic records based on the predefined eligibility criteria and were subsequently approached and invited to participate. No refusals were recorded among the individuals approached, and no participants discontinued questionnaire completion after agreeing to participate. Eligible individuals who were unavailable during the data-collection sessions were not recorded separately; consequently, an overall enrollment rate relative to the entire eligible population could not be reliably calculated retrospectively.

During data preparation, missing values were identified for several criminological variables. Comparative analyses were therefore conducted using complete cases for each variable of interest. Consequently, the sample size varied according to data availability: analyses by offense category included 487 participants, analyses by sentence length included 493 participants, whereas age-based analyses were conducted using the full sample (N = 500).

### 2.3. Ethics and Informed Consent

The present study complied with all legal and institutional requirements established by the Romanian National Administration of Penitentiaries for conducting scientific research within correctional settings.

The study was conducted in accordance with national and international ethical standards for research involving vulnerable populations. Ethical approval was granted by the Ethics Committee of the Romanian Academy (Approval No. 367, 14 December 2023), and authorization to conduct the study was obtained from the Romanian National Administration of Penitentiaries.

Written informed consent was obtained from all participants. They were informed about the voluntary and anonymous nature of their participation, in accordance with the General Data Protection Regulation (GDPR).

Participation in the study was entirely voluntary, and refusal to participate or subsequent withdrawal from the study had no effect on participants’ institutional status. In accordance with the regulations of the Romanian National Administration of Penitentiaries regarding participation in educational, psychological, and research activities, participants received three institutional credits for their participation. The allocation of these credits represented a standard institutional practice and was not contingent upon the content of participants’ responses.

The questionnaires were administered by psychologists employed within the correctional system who had no responsibility for evaluating participants in relation to institutional decision-making. Participants were informed that their responses would be used exclusively for research purposes, treated confidentially, and would not be disclosed to institutional personnel involved in decisions concerning their institutional status.

### 2.4. Psychological Measures

Four standardized psychological instruments were used to assess the variables included in the present study. All instruments were administered in their Romanian-language versions, available through ResearchCentral, a research initiative developed by TestCentral and the Laboratory for Assessment and Individual Differences at the University of Bucharest. The platform provides psychological instruments that have been translated into Romanian or validated for use with the Romanian population.

To assess psychopathy, two complementary instruments were employed in order to capture both the affective–interpersonal and behavioral dimensions of the construct. Previous research has shown that different self-report measures assess partially distinct dimensions of psychopathy and differ with respect to their factorial structure and construct validity (Tsang et al., 2018; Međedović, 2024).

Psychopathy was assessed using the Self-Report Psychopathy Scale–III (SRP-III; Paulhus et al., 2016), a 64-item self-report measure designed to assess the affective–interpersonal and behavioral dimensions of psychopathy. Responses are rated on a 5-point Likert scale ranging from 1 (strongly disagree) to 5 (strongly agree). The instrument comprises four subscales: Interpersonal Manipulation, Callous Affect, Erratic Lifestyle, and Antisocial Behavior. Scores for each subscale were calculated as the mean of the corresponding 16 items after reverse-scored items had been recoded. The total SRP-III score was computed by summing the mean scores of the four subscales, in accordance with the instrument’s scoring guidelines. The four-factor structure and construct validity of the SRP-III have been supported in a large community sample (Gordts et al., 2017).

Psychopathy was also assessed using the Levenson Self-Report Psychopathy Scale (LSRP; Levenson et al., 1995), a 26-item self-report instrument measuring primary and secondary psychopathy. Responses are rated on a 4-point Likert scale ranging from 1 (strongly disagree) to 4 (strongly agree). The instrument includes two subscales corresponding to primary and secondary psychopathy. Subscale scores were calculated by summing responses to the corresponding items after reverse-scored items had been recoded, following the instrument’s scoring guidelines. Previous studies have demonstrated adequate psychometric properties and satisfactory reliability of the LSRP in both community and forensic populations (Redondo et al., 2023; Sellbom, 2011).

Personality was assessed using the Agreeableness and Conscientiousness scales of the International Personality Item Pool (IPIP; Goldberg et al., 2006), each consisting of 10 items. The Romanian adaptation of the IPIP (Iliescu et al., 2015) was used in the present study. Responses were recorded on a 5-point Likert scale, ranging from 1 (strongly disagree) to 5 (strongly agree). Validation studies of the Romanian IPIP have reported satisfactory psychometric properties, including adequate reliability and construct validity (Iliescu et al., 2015).

Sociodemographic and criminological data were collected using a structured questionnaire, including variables such as age, offense type, and sentence length.

### 2.5. Data Analysis

Statistical analyses were performed using IBM SPSS Statistics, Version 27.0 (IBM Corp., Armonk, NY, USA). Descriptive statistics were calculated for all study variables, and the internal consistency of the psychological instruments was assessed using Cronbach’s alpha coefficients and McDonald’s omega coefficients. McDonald’s omega was estimated with the OMEGA macro for SPSS (Hayes & Coutts, 2020), using factor loadings obtained through forced single-factor maximum-likelihood factor analyses.

To test the study hypotheses, one-way analysis of variance (ANOVA), Pearson’s correlation coefficient, Spearman’s rank-order correlation coefficient, and hierarchical linear regression analyses were conducted. The assumption of homogeneity of variances was assessed using Levene’s test. As the test was nonsignificant (*p* > 0.05), indicating that the homogeneity of variance assumption was met, post hoc multiple comparisons were conducted using Tukey’s HSD test.

The selection of candidate variables included in the regression model was based on the literature, which indicates that sociodemographic and criminological factors may be associated with levels of psychopathic traits in incarcerated populations. Previous studies have frequently included variables such as age, sex, educational level, area of residence, offense type, and sentence length in explanatory models of psychopathy (García et al., 2012; de Oliveira et al., 2024). To identify the variables that contributed significantly to explaining psychopathy in the analyzed sample, three predictor-selection procedures (Forward, Stepwise, and Backward) were applied, all of which resulted in the same final model, supporting the stability of covariate selection. The fact that sex was not retained by any of the three selection procedures should be interpreted with caution: although the overall sample size (N = 500) provides adequate power for the main analyses reported, the small size of the female subgroup (n = 25, 5.0% of the sample) substantially limits the power to detect potential sex-based differences specifically, independently of the overall sample size.

We further note, in response to the reviewers, that the use of automated variable-selection procedures (Forward, Stepwise, Backward) carries recognized methodological limitations, including potential instability of coefficient estimates and reduced replicability, and that this stage of the analysis should accordingly be regarded as exploratory rather than confirmatory (Whittingham et al., 2006); convergence of the three procedures on an identical final model provides some indication of stability in the present sample. In addition, Tukey’s HSD test controls the family-wise error rate for the pairwise comparisons conducted within each ANOVA; no additional correction was applied across the separate ANOVA models (by offense type, age group, and sentence length category) or across the partially overlapping dependent variables, consistent with recommendations that such correction is most clearly warranted in confirmatory designs testing a single combined hypothesis rather than in largely descriptive, multi-outcome comparisons such as the present one (Bender & Lange, 2001); this should be kept in mind when interpreting the smaller and more marginal effects reported.

The study’s hypotheses are presented in Section 1 (Introduction).

## 3. Results

### Descriptive Statistics

Participants were classified into four age groups, with the largest proportion belonging to the 31–40 years age group (38.4%), followed by the 41–50 years (25.2%), 21–30 years (24.0%), and over 50 years (12.4%) age groups.

Regarding place of residence, the majority of participants originated from urban areas (68.4%). Educational attainment was generally low, with 72.0% of participants having completed no more than lower secondary education.

With respect to criminal history, 57.6% of participants were serving their first custodial sentence. Information on offense category was available for 487 participants, with missing data for 13 cases (2.6%). Among these participants, physical violence offenses were the most frequent (54.8%), followed by offenses against life (27.7%) and sexual violence offenses (17.5%). The distribution of participants according to offense type and sentence length is presented in Table 1.

The internal consistency of the study measures is presented in Table 2. Cronbach’s alpha and McDonald’s omega coefficients indicated good internal consistency for the total scores of the Levenson Self-Report Psychopathy Scale (LSRP; α = 0.845, ω = 0.853) and the Self-Report Psychopathy Scale–III (SRP-III; α = 0.846, ω = 0.841), as well as for the Conscientiousness scale (α = 0.801, ω = 0.786). The LSRP subscales also showed satisfactory internal consistency (Primary Psychopathy: α = 0.790, ω = 0.784; Secondary Psychopathy: α = 0.718, ω = 0.732). In contrast, reliability estimates for the SRP-III subscales were lower and more variable than those for the overall scale (Interpersonal Manipulation: α = 0.657, ω = 0.642; Callous Affect: α = 0.535, ω = 0.010; Erratic Lifestyle: α = 0.608, ω = 0.601; Antisocial Behavior: α = 0.568, ω = 0.397). Agreeableness showed α = 0.744 and ω = 0.729.

The exceptionally low McDonald’s omega coefficient for the Callous Affect subscale (ω = 0.010) should be interpreted in the context of the forced single-factor model used for its estimation. The corresponding factor solution yielded item loadings in opposite directions, suggesting that a single-factor omega estimate may not adequately represent the internal structure of this subscale in the present sample.

Descriptive statistics for the study variables are presented in Table 3. The means and standard deviations indicated moderate variability across the psychopathy dimensions and personality traits examined. Skewness and kurtosis values were within acceptable ranges, suggesting that the variables were approximately normally distributed and supporting the use of parametric statistical analyses.

Correlations among the study variables are presented in Table 4. The results revealed a significant positive correlation between the overall psychopathy scores obtained with the SRP-III and the LSRP (r = 0.65, *p* < 0.01), indicating a moderately strong association between the two measures.

Furthermore, the psychopathy dimensions were significantly and negatively correlated with the personality traits of agreeableness and conscientiousness, indicating that higher levels of psychopathy were associated with lower levels of these personality traits.

Spearman’s rank-order correlations were additionally calculated for age category and sentence-length category because these variables were ordinal. Increasing age category was significantly associated with lower scores on several psychopathy measures, with coefficients ranging from ρ = −0.091 to ρ = −0.206. A similar pattern was observed for sentence-length category, which showed small negative associations with several psychopathy measures, with the strongest association observed for Erratic Lifestyle (ρ = −0.206, *p* < 0.01). Age category and sentence-length category were positively associated (ρ = 0.265, *p* < 0.01). Overall, these findings indicate modest monotonic trends across the ordered age and sentence-length categories.

As a supplementary analysis, the correlations presented in Table 4 were examined separately for men and women. The overall pattern of associations among the psychopathy measures was broadly similar across the two groups, with strong positive correlations among related psychopathy dimensions. However, some descriptive differences were observed. The negative associations between Agreeableness and Conscientiousness and psychopathy measures were more consistent among men, whereas these associations were weaker and largely non-significant among women. Some associations involving Antisocial Behavior also appeared stronger among women. Given the small number of women in the sample (n = 25), these subgroup-specific findings should be interpreted cautiously and are presented descriptively rather than as evidence of statistically established sex differences.

Differences according to violent offense type were examined using one-way analysis of variance (ANOVA) based on the 487 participants for whom offense category data were available. The results revealed significant differences in the total psychopathy scores measured by the LSRP, F(2, 484) = 9.75, *p* < 0.001, η^2^ = 0.039, as well as in the SRP-III total scores, F(2, 484) = 12.13, *p* < 0.001, η^2^ = 0.048.

Significant group differences were also observed for the SRP-III subscales of Interpersonal Manipulation, F(2, 484) = 7.60, *p* < 0.01, η^2^ = 0.030, Erratic Lifestyle, F(2, 484) = 14.50, *p* < 0.001, η^2^ = 0.057, and Antisocial Behavior, F(2, 484) = 14.49, *p* < 0.001, η^2^ = 0.057.

Post hoc pairwise comparisons were conducted using Tukey’s HSD test, as described in Section 2.5. Post hoc comparisons indicated that participants convicted of physical violence offenses obtained significantly higher psychopathy scores than those convicted of offenses against life or sexual violence offenses. The means and standard deviations for each offense category are presented in Table 5.

Differences in psychopathy dimensions across age groups were examined using one-way analysis of variance (ANOVA). The results revealed significant differences in the total psychopathy scores measured by the LSRP, F(3, 496) = 2.95, *p* < 0.05, η^2^ = 0.018, as well as in the SRP-III total scores, F(3, 496) = 4.16, *p* < 0.01, η^2^ = 0.025.

Significant group differences were also observed for the SRP-III subscales of Interpersonal Manipulation, F(3, 496) = 2.75, *p* < 0.05, η^2^ = 0.016, Erratic Lifestyle, F(3, 496) = 7.78, *p* < 0.001, η^2^ = 0.045, and Antisocial Behavior, F(3, 496) = 2.59, *p* < 0.05, η^2^ = 0.015.

Post hoc comparisons indicated that participants aged over 50 years obtained significantly lower psychopathy scores than those aged 21–30 years. Overall, the findings suggest that older participants tended to report lower levels of psychopathy than younger participants. The means and standard deviations for each age group are presented in Table 6.

In addition to the primary analyses, differences according to sentence length were also examined, given the criminological relevance of this variable and its potential association with psychopathy dimensions. The analysis included 493 participants for whom complete information on sentence length was available.

The results revealed significant differences in the SRP-III total psychopathy scores, F(2, 490) = 4.57, *p* < 0.05, η^2^ = 0.018, as well as for the SRP-III subscales of Interpersonal Manipulation, F(2, 490) = 3.88, *p* < 0.05, η^2^ = 0.016, and Erratic Lifestyle, F(2, 490) = 11.71, *p* < 0.001, η^2^ = 0.046.

Post hoc comparisons indicated that participants serving shorter sentences obtained significantly higher psychopathy scores than those serving longer sentences. The means and standard deviations for each sentence-length category are presented in Table 7.

A hierarchical linear regression analysis was conducted to examine the contribution of personality traits to explaining psychopathy levels. In the hierarchical regression analysis, the SRP-III total score was used as the dependent variable because it provides an integrated multidimensional assessment of psychopathy, encompassing interpersonal, affective, behavioral, and antisocial dimensions. This choice was consistent with the study’s objective of investigating the role of personality traits in explaining overall psychopathy levels.

Following the selection procedures described in the Data Analysis section, age group and sentence length category were retained as control variables. The control model explained 3% of the variance in psychopathy levels (*R*^2^ = 0.03, *F*(2, 490) = 7.74, *p* < 0.001).

Adding Agreeableness and Conscientiousness in the second step of the hierarchical regression significantly improved the explanatory power of the model (ΔR^2^ = 0.10, F_change_(2, 488) = 26.62, *p* < 0.001), with the final model accounting for 13% of the variance in psychopathy (R^2^ = 0.13, F(4, 488) = 17.58, *p* < 0.001).

Both personality traits emerged as significant negative predictors of psychopathy, with Agreeableness (β = −0.179, *p* < 0.001) and Conscientiousness (β = −0.175, *p* = 0.001) being associated with lower psychopathy scores. In the final model, age group remained a significant predictor (β = −0.112, *p* = 0.012), whereas sentence length category was no longer a statistically significant predictor of psychopathy (β = −0.072, *p* = 0.106). The regression coefficients are presented in Table 8.

## 4. Discussion

Understanding the psychological mechanisms underlying differences in violent behavior represents a central focus of contemporary criminological psychology. Although psychopathy is frequently conceptualized as a major explanatory factor for violent behavior, empirical evidence indicates that it is not a unitary construct but rather a multidimensional one, whose manifestations vary according to the offending context and individual characteristics. Distinct subtypes of psychopathic offenders have even been identified (J. Skeem et al., 2007). Within this context, the present study contributes to the literature by highlighting differences in the expression of psychopathy across types of violent offenses and by clarifying the role of personality traits in shaping the psychopathic profile within correctional settings.

Recent research has provided increasing support for the heterogeneous nature of psychopathy, highlighting the existence of distinct psychopathic profiles characterized by different combinations of affective, interpersonal, and behavioral traits. For example, latent profile analyses have identified substantial variation in the manifestation of psychopathic characteristics, suggesting that not all individuals with elevated psychopathy scores exhibit the same pattern of emotional functioning, behavioral regulation, or risk for violent behavior (Moffett et al., 2020). Similarly, contemporary reviews emphasize that psychopathy should be understood as a multidimensional construct comprising affective, interpersonal, behavioral, and antisocial dimensions, the expression of which may vary according to both individual and contextual characteristics (Sellbom & Drislane, 2021; De Brito et al., 2021).

The importance of examining psychopathy dimensions separately is further supported by recent research demonstrating that scores on the SRP-III facets provide more accurate predictive information than the overall psychopathy score with respect to different forms of criminal and deviant behavior. These findings underscore the value of examining the individual dimensions of psychopathy rather than relying exclusively on the global score (Gabriel & Renaud, 2024).

The findings support the first hypothesis, indicating that the affective–interpersonal and behavioral dimensions of psychopathy differ according to the type of violent offense committed. Higher levels of the behavioral dimensions were observed among participants convicted of physical violence offenses, suggesting that this group exhibits a more pronounced expression of the behavioral components of psychopathy.

These findings are consistent with previous research demonstrating associations between the behavioral dimensions of psychopathy—characterized by impulsivity, sensation seeking, and reduced behavioral control—and higher levels of aggression and violent behavior (Leistico et al., 2008; Thomson et al., 2025). Individuals exhibiting elevated levels of behavioral psychopathic traits may be more likely to engage in direct acts of violence because of difficulties in emotional regulation, reduced inhibitory control, and an increased tendency to respond impulsively to perceived threats or frustration.

These findings challenge the assumption that psychopathy is expressed uniformly and are consistent with previous research demonstrating considerable variability in the relationship between psychopathic traits and violent behavior (Walsh et al., 2007; J. L. Skeem et al., 2011). The higher levels of the behavioral dimensions of psychopathy observed among participants convicted of physical violence offenses suggest a greater involvement of impulsivity, deficits in behavioral regulation, and characteristics of an antisocial lifestyle in direct forms of violent behavior. This interpretation is supported by recent empirical evidence indicating that the behavioral manifestations of psychopathy are more strongly associated with aggressive behavior, impaired self-regulation, and an increased propensity for direct violent acts (Moffett et al., 2020; Thomson et al., 2025).

The distinction between instrumental and reactive violence provides a relevant theoretical framework for interpreting the relationship between psychopathy and violent behavior. Instrumental violence is goal-directed and typically involves a higher degree of planning, whereas reactive violence occurs primarily in response to perceived provocation or frustration and is characterized by heightened emotional arousal (Woodworth & Porter, 2002). Previous research has linked psychopathy to both forms of violence, although the association with instrumental violence has been particularly evident in some forensic samples. In the present study, offenses were classified according to legal offense categories, without assessing the underlying motivation, degree of planning, or emotional context of the offense. Consequently, the higher behavioral psychopathy scores observed among participants convicted of physical violence offenses do not allow conclusions regarding whether these offenses were instrumental or reactive but rather indicate an association with more direct forms of violent behavior.

The second hypothesis, predicting differences in psychopathy according to age, was supported. The findings indicate that the behavioral dimensions of psychopathy tend to decline with increasing age. This pattern is consistent with longitudinal research demonstrating reductions in impulsivity and antisocial behavior across adulthood (Luengo et al., 1994; Loeber et al., 2012). Previous studies suggest that the behavioral manifestations of psychopathy are more susceptible to developmental change than the affective–interpersonal components, although the literature also indicates that certain interpersonal dimensions may vary as a function of age (Blonigen, 2010; Hare & Neumann, 2008). The decline observed in the present study may reflect improvements in behavioral control with age, reduced sensation seeking, and a gradual decrease in engagement in risky and antisocial behaviors. These findings are consistent with developmental criminology perspectives, which propose that antisocial behavior generally declines with age, even among individuals exhibiting elevated levels of psychopathic traits.

Within correctional settings, younger incarcerated individuals tend to exhibit higher levels of these behavioral manifestations than older inmates (Porter et al., 2001). These findings support the notion of a differentiated developmental trajectory of psychopathy, in which the behavioral dimensions appear to be more sensitive to developmental influences than the affective–interpersonal dimensions. However, the results of the present study also indicate that interpersonal manipulation varies across age groups, suggesting that certain interpersonal components are not entirely independent of developmental processes. This pattern is consistent with research indicating that the age-related decline in antisocial behavior is explained primarily by reductions in impulsivity, risk-taking, and deficits in behavioral regulation rather than by substantial changes in core affective traits.

The third hypothesis, concerning the predictive role of personality traits, was supported. Agreeableness and Conscientiousness emerged as significant negative predictors of psychopathy, even after controlling for sociodemographic and criminological variables. These findings are consistent with previous research identifying low levels of Agreeableness and Conscientiousness as fundamental correlates of psychopathic traits (Lynam & Miller, 2019; Decuyper et al., 2009). More specifically, low Agreeableness reflects antagonistic interpersonal tendencies, reduced empathy, and hostility, whereas low Conscientiousness is associated with impulsivity, poor behavioral regulation, and reduced self-control—characteristics that substantially overlap with the core dimensions of psychopathy (Ross et al., 2004; Van Til et al., 2022).

Importantly, the findings suggest that these personality dimensions contribute to explaining variation in psychopathy beyond the influence of sociodemographic and criminological characteristics. This result highlights the relevance of dispositional factors in understanding individual differences in psychopathic traits among individuals convicted of violent offenses and supports the inclusion of personality assessment in psychological evaluation protocols within correctional settings. Incorporating personality dimensions such as Agreeableness and Conscientiousness into psychological assessments may improve risk appraisal and facilitate the development of intervention and rehabilitation strategies that are better tailored to the individual characteristics of incarcerated persons. Nevertheless, the proportion of variance explained by the final model indicates that psychopathy is also influenced by additional individual and contextual factors that were not included in the present analysis.

The present findings are consistent with research integrating the Five-Factor Model into explanations of antisocial behavior, demonstrating robust negative associations between psychopathy and both Agreeableness and Conscientiousness (Paulhus & Williams, 2002; Ross et al., 2004; Sadeh & Verona, 2008; Van Til et al., 2022). These relationships have been consistently supported by meta-analytic evidence (Decuyper et al., 2009), including studies examining aggressive behavior (Xu et al., 2024). From a psychological perspective, low Agreeableness reflects interpersonal antagonism and deficits in empathy (Graziano et al., 2007; Lynam & Miller, 2019), a relationship further supported by the meta-analysis conducted by Burghart and Mier (2022), which reported consistent associations between psychopathy and reduced affective empathy. In contrast, low Conscientiousness is indicative of impulsivity and poor behavioral regulation (Ross et al., 2004; Settles et al., 2012). These characteristics largely overlap with the core dimensions of psychopathy and may help explain the negative associations observed in the present study. Consistent with this interpretation, recent research on the Dark Triad has demonstrated distinct mechanisms through which these traits contribute to aggressive behavior, with psychopathy emerging as a significant predictor of physical aggression and anger, whereas Machiavellianism was more strongly associated with verbal aggression and hostility (Rada & Forțu, 2026).

Taken together, these findings indicate that higher levels of Agreeableness and Conscientiousness are associated with lower levels of psychopathic traits and with less pronounced behavioral and interpersonal characteristics associated with psychopathy.

Overall, the findings support the view that individual characteristics and contextual factors should be considered in an integrated manner. The effects of the correctional environment cannot be understood solely in terms of institutional characteristics but must also be interpreted in light of the psychological characteristics of incarcerated individuals (Boduszek et al., 2016).

### Limitations and Future Research

The findings should be interpreted in light of several methodological limitations. First, the use of self-report measures in correctional settings may be affected by impression management, the tendency to minimize problematic behaviors, and the institutional context in which the assessments were conducted. Completing the questionnaires in the presence of a representative of the correctional system may have influenced participants’ openness and candor, thereby limiting control over the validity of their responses. Furthermore, although the use of two self-report instruments enabled the assessment of complementary dimensions of psychopathy, the absence of clinician-administered measures, such as the Psychopathy Checklist–Revised (PCL-R), may limit the comparability of the findings with studies employing forensic assessments.

A second limitation concerns the institutional credits awarded for participation in the study. Although these credits were granted in accordance with the regulations of the Romanian National Administration of Penitentiaries and represent standard institutional practice for participation in educational, psychological, and research activities, it cannot be ruled out that they may have influenced some participants’ willingness to take part in the study. However, the credits were awarded solely for participation and were not contingent upon the content of participants’ responses.

In addition, the cross-sectional design precludes causal inferences regarding the relationships among the variables examined. Consequently, the findings should be interpreted as reflecting associations among psychopathy dimensions, personality traits, and criminological variables. Furthermore, the absence of a comparison group consisting of individuals convicted of non-violent offenses or members of the general population limits the ability to determine whether the observed patterns are specific to individuals convicted of violent offenses or reflect broader characteristics of incarcerated populations. The small proportion of women in the sample (5.0%), although representative of the gender distribution within the Romanian prison population, also limits the generalizability of the findings to incarcerated women. Sex-stratified correlation analyses suggested a broadly similar pattern of associations among psychopathy measures, but also indicated some descriptive differences, particularly in the associations of psychopathy with Agreeableness, Conscientiousness, and Antisocial Behavior. Given the small female subgroup (n = 25), however, these differences cannot be interpreted as robust evidence of sex-specific patterns and should be examined in future studies using larger and more balanced samples.

Participants were recruited from six correctional facilities that varied in institutional profile, including large-capacity prisons, a prison hospital, and a facility exclusively for female inmates. Although the present design does not model this clustering explicitly, organizational and contextual characteristics specific to each facility (e.g., security regime, staff practices, population composition) may have influenced participants’ responses independently of the individual-level variables examined, introducing a potential source of non-independence in the data that was not accounted for in the present analyses. Future studies with adequate facility-level sample sizes should consider multilevel modeling to formally partition variance attributable to the correctional facility.

The selection of control variables was conducted using an exploratory procedure involving the Forward, Stepwise, and Backward methods. Although all three procedures resulted in the same final model, suggesting stability in the selection process, the data-driven nature of this stage represents a methodological limitation. Therefore, the role of the identified covariates should be confirmed in independent samples using models specified *a priori*.

Although most of the observed differences reached statistical significance, the η^2^ values indicated predominantly small effect sizes. Given the large sample size, some differences of limited magnitude may have achieved statistical significance. Therefore, the findings should be interpreted not only in terms of statistical significance but also with regard to their practical significance.

Interpreted against contemporary benchmarks for effect sizes in psychological research, the associations reported here—corresponding to correlations in the small range—are consistent with effects that, while modest at the level of a single individual, can be practically meaningful when aggregated across a large correctional population or applied cumulatively across multiple assessment domains (Funder & Ozer, 2019). In practice, this suggests that offense type, age, and the personality traits examined should be regarded as contributing factors within a broader, multi-component risk-assessment framework, rather than as sufficient grounds, on their own, for individual-level clinical or judicial decisions.

The SRP-III subscales showed lower and more variable internal consistency than the total score, particularly Callous Affect (α = 0.535, ω = 0.010), while Interpersonal Manipulation (α = 0.657, ω = 0.642), Erratic Lifestyle (α = 0.608, ω = 0.601), and Antisocial Behavior (α = 0.568, ω = 0.397) also yielded coefficients below conventional reliability thresholds. The low reliability of these facet-level scores may have attenuated observed associations and effect-size estimates; this is particularly relevant for Callous Affect, which was the only SRP-III dimension that did not yield significant differences in the analyses. Accordingly, findings involving the SRP-III subscales should be interpreted with caution and regarded as preliminary until replicated using measures with stronger facet-level reliability.

A further limitation concerns the absence of representative Romanian general-population normative data directly comparable across the psychopathy and personality measures used in the present study. Although descriptive values have been reported in international community samples and in Romanian adaptation research for some of these measures, these samples cannot be assumed to constitute population norms. Consequently, direct statistical comparisons with the general population were not performed, as such comparisons could confound population differences with cultural, demographic, and sampling differences.

Future research would benefit from longitudinal designs examining changes in psychopathy dimensions over the course of incarceration and investigating the relationships among personality traits, coping strategies, criminological variables, and characteristics of the correctional environment. In addition, the use of multimethod assessment approaches combining self-report measures with behavioral and clinician-administered assessments could enhance the validity of the findings and contribute to the development of more comprehensive explanatory models of adjustment to correctional settings and violent behavior. Future studies should also examine the role of behavioral coping strategies as a potential mediator of the relationship between psychopathic traits and both adjustment to correctional settings and violent behavior. Finally, including comparison groups composed of individuals convicted of non-violent offenses or members of the general population would help determine the extent to which the patterns identified in the present study are specific to violent offenders.

## 5. Conclusions

The present study examined how psychopathy dimensions differ according to the type of violent offense, age, and personality traits in a sample of 500 incarcerated individuals from six correctional institutions in Romania. The findings support the view that psychopathy, as assessed within correctional settings, is best understood as a multidimensional rather than a unitary construct, with its behavioral dimensions varying systematically according to both the type of violent offense and age. Furthermore, Agreeableness and Conscientiousness contributed significantly to explaining variation in psychopathy beyond the effects of sociodemographic and criminological variables.

These findings have both theoretical and practical implications. From a theoretical perspective, the study provides empirical evidence from a national context that has received limited attention in the literature, supporting a dimensional conceptualization of psychopathy. The findings suggest that relying solely on global psychopathy scores may obscure important differences between the affective–interpersonal and behavioral dimensions of the construct—differences that are directly relevant to understanding the heterogeneity of violent behavior. From an applied perspective, the results suggest that the psychological assessment of individuals convicted of violent offenses may benefit from integrating dimensional measures of psychopathy with the assessment of personality traits such as Agreeableness and Conscientiousness. Such an approach may facilitate more nuanced risk appraisal and support the development of intervention strategies tailored to the individual profiles of incarcerated persons.

At the same time, the predominantly small effect sizes observed indicate that psychopathy cannot be understood solely in terms of the variables examined in the present study but is likely to reflect the combined influence of dispositional, criminological, and contextual factors, including institutional and interpersonal influences, that warrant further investigation through longitudinal and multimethod research. In this respect, the present study offers a foundation for a more comprehensive approach to the assessment of psychopathy within Romanian correctional settings—one that moves beyond reliance on global psychopathy scores and emphasizes both the multidimensional nature of psychopathy and the contribution of personality characteristics to understanding the psychological profiles of individuals convicted of violent offenses.

## Figures and Tables

**Table 1:** Distribution of Participants by Offense Type and Sentence Length.

Offense Type	Short (8–60 Months) n (%)	Medium (61–120 Months) n (%)	Long (>120 Months) n (%)	Missing n (%)	Total n (%)
Offenses against life	13 (9.6)	22 (16.3)	96 (71.1)	4 (3.0)	135 (27.7)
Physical violence offenses	177 (66.3)	70 (26.2)	20 (7.5)	0 (0.0)	267 (54.8)
Sexual violence offenses	31 (36.5)	40 (47.1)	13 (15.3)	1 (1.2)	85 (17.5)

Note. Values are presented as n (%).

**Table 2:** Internal Consistency of the Study Scales and Subscales.

Scale/Subscale	Items (n)	Cronbach’s α	95% CI for Cronbach’s α	McDonald’s ω
LSRP Total Psychopathy	26	0.845	0.824–0.864	0.853
Primary Psychopathy	16	0.790	0.762–0.816	0.784
Secondary Psychopathy	10	0.718	0.679–0.753	0.732
SRP-III Total Psychopathy	64	0.846	0.826–0.864	0.841
Interpersonal Manipulation	16	0.657	0.611–0.699	0.642
Callous Affect	16	0.535	0.474–0.593	0.010
Erratic Lifestyle	16	0.608	0.556–0.657	0.601
Antisocial Behavior	16	0.568	0.511–0.621	0.397
Agreeableness (IPIP)	10	0.744	0.709–0.776	0.729
Conscientiousness (IPIP)	10	0.801	0.774–0.826	0.786

**Table 3:** Descriptive Statistics of the Study Variables.

Variable	N	M	SD	Median	Skewness	Kurtosis
LSRP Total Psychopathy	500	56.14	11.65	55.00	0.16	−0.52
Primary Psychopathy	500	34.95	7.96	34.00	0.15	−0.79
Secondary Psychopathy	500	21.19	5.07	20.00	0.24	−0.52
SRP-III Total Psychopathy	500	10.14	1.74	10.13	−0.01	0.13
Interpersonal Manipulation	500	2.43	0.56	2.44	0.15	−0.19
Callous Affect	500	2.48	0.49	2.44	0.19	−0.01
Erratic Lifestyle	500	2.57	0.55	2.56	0.18	0.01
Antisocial Behavior	500	2.66	0.55	2.69	−0.29	0.21
Agreeableness (IPIP)	500	37.87	6.22	38.00	−0.19	−0.19
Conscientiousness (IPIP)	500	38.51	6.82	39.00	−0.40	−0.45

Note. *M* = mean; *SD* = standard deviation.

**Table 4:** Correlations Among the Study Scales and Subscales.

Variable	1	2	3	4	5	6	7	8	9	10	11
1. LSRP Total Psychopathy	—										
2. Primary Psychopathy	0.94 **	—									
3. Secondary Psychopathy	0.83 **	0.58 **	—								
4. SRP-III Total Psychopathy	0.65 **	0.63 **	0.50 **	—							
5. Interpersonal Manipulation	0.61 **	0.61 **	0.43 **	0.87 **	—						
6. Callous Affect	0.57 **	0.56 **	0.44 **	0.79 **	0.66 **	—					
7. Erratic Lifestyle	0.53 **	0.49 **	0.45 **	0.84 **	0.63 **	0.57 **	—				
8. Antisocial Behavior	0.39 **	0.39 **	0.29 **	0.75 **	0.53 **	0.38 **	0.51 **	—			
9. Agreeableness	−0.34 **	−0.33 **	−0.26 **	−0.28 **	−0.28 **	−0.31 **	−0.19 **	−0.13 **	—		
10. Conscientiousness	−0.30 **	−0.25 **	−0.28 **	−0.27 **	−0.27 **	−0.24 **	−0.28 **	−0.13 **	0.53 **	—	
11. Age Category	−0.113 *	−0.105 *	−0.107 *	−0.130 **	−0.091 *	−0.042	−0.206 **	−0.080	0.029	0.064	—
12. Sentence-Length Category	−0.104 *	−0.091 *	−0.096 *	−0.121 **	−0.115 *	−0.027	−0.206 **	−0.063	0.066	0.113 *	0.265 **

Note. Pearson correlation coefficients (*r*) are reported for associations among the psychological variables (Variables 1–10). Spearman’s rank-order correlation coefficients (*ρ*) are reported for associations involving Age Category and Sentence-Length Category (Variables 11–12). LSRP = Levenson Self-Report Psychopathy Scale; SRP-III = Self-Report Psychopathy Scale–III. * *p* < 0.05; ** *p* < 0.01 (two-tailed).

**Table 5:** Psychopathy Scores by Type of Violent Offense.

Variable	Offenses Against Life		Sexual Violence Offenses		Physical Violence Offenses		*F* (2, 484)	η^2^
	M	SD	M	SD	M	SD		
LSRP Total Psychopathy	53.36 ^a^	11.38	54.11 ^a^	11.72	58.24 ^b^	11.50	9.75 ***	0.039
Primary Psychopathy	33.30 ^a^	7.46	33.11 ^a^	8.11	36.31 ^b^	7.91	9.40 ***	0.037
Secondary Psychopathy	20.05 ^a^	5.31	21.00 ^ab^	4.87	21.93 ^b^	4.88	6.44 **	0.026
SRP-III Total Psychopathy	9.77 ^a^	1.73	9.64 ^a^	1.73	10.48 ^b^	1.69	12.13 ***	0.048
Interpersonal Manipulation	2.35 ^a^	0.55	2.30 ^a^	0.54	2.52 ^b^	0.55	7.60 **	0.030
Callous Affect	2.44 ^a^	0.49	2.44 ^a^	0.50	2.51 ^a^	0.49	1.08	0.004
Erratic Lifestyle	2.41 ^a^	0.57	2.47 ^a^	0.46	2.69 ^b^	0.54	14.50 ***	0.057
Antisocial Behavior	2.57 ^a^	0.57	2.44 ^a^	0.52	2.77 ^b^	0.52	14.49 ***	0.057

Note. *M* = mean; *SD* = standard deviation; *F* = one-way analysis of variance (ANOVA); η^2^ = eta squared. ** *p* < 0.01; *** *p* < 0.001. Means that do not share the same superscript letter differ significantly according to Tukey’s HSD test (*p* < 0.05).

**Table 6:** Comparison of Psychopathy Dimensions Across Age Groups.

Variable	21–30 Years		31–40 Years		41–50 Years		Over 50 Years		*F*(3, 496)	η^2^
	M	SD	M	SD	M	SD	M	SD		
LSRP Total Psychopathy	57.64 ^a^	10.97	56.74 ^a^	12.28	55.58 ^ab^	11.54	52.53 ^b^	10.56	2.95 *	0.018
Primary Psychopathy	35.78 ^a^	7.53	35.47 ^ab^	8.53	34.42 ^ab^	7.57	32.81 ^b^	7.41	2.41	0.014
Secondary Psychopathy	21.87 ^a^	4.89	21.27 ^ab^	5.18	21.16 ^ab^	5.30	19.73 ^b^	4.32	2.47	0.015
SRP-III Total Psychopathy	10.34 ^a^	1.82	10.30 ^a^	1.69	9.95 ^ab^	1.75	9.54 ^b^	1.59	4.16 **	0.025
Interpersonal Manipulation	2.46 ^a^	0.61	2.50 ^ab^	0.52	2.38 ^ab^	0.54	2.29 ^b^	0.56	2.75 *	0.016
Callous Affect	2.50 ^a^	0.48	2.49 ^a^	0.51	2.47 ^a^	0.51	2.40 ^a^	0.44	0.61	0.004
Erratic Lifestyle	2.70 ^a^	0.57	2.62 ^ab^	0.52	2.46 ^bc^	0.54	2.36 ^c^	0.50	7.78 ***	0.045
Antisocial Behavior	2.69 ^a^	0.55	2.70 ^a^	0.54	2.65 ^ab^	0.55	2.48 ^b^	0.52	2.59 *	0.015

Note. *M* = mean; *SD* = standard deviation; *F* = one-way analysis of variance (ANOVA); η^2^ = eta squared. * *p* < 0.05; ** *p* < 0.01; *** *p* < 0.001. Means that do not share the same superscript letter differ significantly according to Tukey’s HSD test (*p* < 0.05).

**Table 7:** Comparison of Psychopathy Dimensions Across Sentence Length Categories.

Variable	Short Sentence		Medium Sentence		Long Sentence		*F*(2, 490)	η^2^
	M	SD	M	SD	M	SD		
LSRP Total Psychopathy	57.39 ^a^	11.72	55.41 ^a^	11.33	54.60 ^a^	11.64	2.72	0.011
Primary Psychopathy	35.74 ^a^	8.06	34.36 ^a^	7.96	34.07 ^a^	7.65	2.29	0.009
Secondary Psychopathy	21.65 ^a^	4.94	21.05 ^a^	4.97	20.53 ^a^	5.32	2.08	0.008
SRP-III Total Psychopathy	10.36 ^a^	1.77	10.16 ^ab^	1.67	9.78 ^b^	1.65	4.57 *	0.018
Interpersonal Manipulation	2.51 ^a^	0.58	2.41 ^ab^	0.51	2.34 ^b^	0.55	3.88 *	0.016
Callous Affect	2.49 ^a^	0.50	2.47 ^a^	0.50	2.47 ^a^	0.48	0.17	0.001
Erratic Lifestyle	2.67 ^a^	0.56	2.58 ^a^	0.50	2.39 ^b^	0.50	11.71 ***	0.046
Antisocial Behavior	2.68 ^a^	0.55	2.70 ^a^	0.56	2.58 ^a^	0.52	1.76	0.007

Note. *M* = mean; *SD* = standard deviation; *F* = one-way analysis of variance (ANOVA); η^2^ = eta squared. * *p* < 0.05; *** *p* < 0.001. Means that do not share the same superscript letter differ significantly according to Tukey’s HSD test (*p* < 0.05).

**Table 8:** Hierarchical Linear Regression Predicting Psychopathy (SRP-III) from Agreeableness and Conscientiousness After Controlling for Sociodemographic and Criminological Variables.

Variable	B	95% CI		SE	β	R^2^	ΔR^2^
		Lower	Upper				
Step 1						0.03	0.03 ***
Age Group	−0.212	−0.376	−0.048	0.083	−0.118 *		
Sentence Length Category	−0.210	−0.400	−0.020	0.097	−0.101 *		
Step 2						0.13	0.10 ***
Age Group	−0.201	−0.357	−0.045	0.079	−0.112 *		
Sentence Length Category	−0.150	−0.331	0.032	0.092	−0.072		
Agreeableness	−0.050	−0.077	−0.023	0.014	−0.179 ***		
Conscientiousness	−0.044	−0.069	−0.019	0.013	−0.175 **		

Note. *B* = unstandardized regression coefficient; *CI* = confidence interval; *SE* = standard error; β = standardized regression coefficient; *R*^2^ = coefficient of determination; Δ*R*^2^ = change in *R*^2^. * *p* < 0.05; ** *p* < 0.01; *** *p* < 0.001.

## Data Availability

Data are available upon reasonable request.
